# Mask-Type Sensor for Pulse Wave and Respiration Measurements and Eye Blink Detection

**DOI:** 10.3390/s21144895

**Published:** 2021-07-19

**Authors:** Thanh-Vinh Nguyen, Masaaki Ichiki

**Affiliations:** Sensing System Research Center, National Institute of Advanced Industrial Science and Technology (AIST), Tsukuba 305-8564, Ibaraki, Japan; ichiki-m@aist.go.jp

**Keywords:** pulse wave, respiration, eye blink, mask, microelectromechanical system, piezoresistive, cantilever

## Abstract

This paper reports on a mask-type sensor for simultaneous pulse wave and respiration measurements and eye blink detection that uses only one sensing element. In the proposed sensor, a flexible air bag-shaped chamber whose inner pressure change can be measured by a microelectromechanical system-based piezoresistive cantilever was used as the sensing element. The air bag-shaped chamber is fabricated by wrapping a sponge pad with plastic film and polyimide tape. The polyimide tape has a hole to which the substrate with the piezoresistive cantilever adheres. By attaching the sensor device to a mask where it contacts the nose of the subject, the sensor can detect the pulses and eye blinks of the subject by detecting the vibration and displacement of the nose skin caused by these physiological parameters. Moreover, the respiration of the subject causes pressure changes in the space between the mask and the face of the subject as well as slight vibrations of the mask. Therefore, information about the respiration of the subject can be extracted from the sensor signal using either the low-frequency component (<1 Hz) or the high-frequency component (>100 Hz). This paper describes the sensor fabrication and provides demonstrations of the pulse wave and respiration measurements as well as eye blink detection using the fabricated sensor.

## 1. Introduction

Wearable devices that can measure physiological parameters such as pulse waves, respiration, and eye blinks are important in various applications, including medical diagnosis, sports, physical training, and health monitoring of workers, drivers, etc. For example, pulse waves can be used as an indicator to diagnose cardiovascular diseases and diabetes [1,2,3], whereas information on respiration is useful for monitoring chronic respiratory diseases such as asthma and chronic obstructive pulmonary disease [4]. Meanwhile, eye blink detection has several uses such as in the evaluation of drowsiness [5] and the diagnosis of dry eye disease [6]. In particular, during and after the current COVID-19 era, wearable devices for physiological monitoring are expected to be useful in mitigating this disease [7,8]. For example, changes in physiological parameters such as heart and respiration rates can be used for the early detection of COVID-19, which is extremely important in preventing the spread of this disease [9,10,11]. Therefore, the need for devices to perform continuous monitoring of physiological parameters in not only hospitals but also in home settings has been increasing rapidly [12].

To realize the long-term measurement of the physiological parameters, it is essential to recognize wearable sensors that can cause minimal discomfort to subjects. Consequently, sensors that can simultaneously measure multiple physiological parameters using a single sensing element are highly desired because they will reduce the number of devices attached to the body of the subject. However, in most commercialized wearable devices for physiological monitoring, each sensor can usually provide information about only one physiological parameter. Thus, to realize simultaneous measurements of multiple physiological parameters, it is often necessary to use multiple sensors simultaneously. Recently, our group reported a sensor type of eyeglasses that could simultaneously measure pulse waves and respiration using a single sensing element [13]. In this device, a tube-shaped pressure sensor was attached to the nose pad of eyeglasses and the pulse wave and respiration of the subject were extracted as low frequency (<10 Hz) and high frequency (>100 Hz) components of the sensor signal, respectively. Besides the ability to measure pulse waves and respiration simultaneously, this device also has advantages such as a simple structure and a simple data processing algorithm, which are favorable for the commercialization of the sensor. However, as the sensor detects respiration by detecting vibrations of the nose skin caused by the breath, it cannot detect mouth breathing.

**Figure 1:**
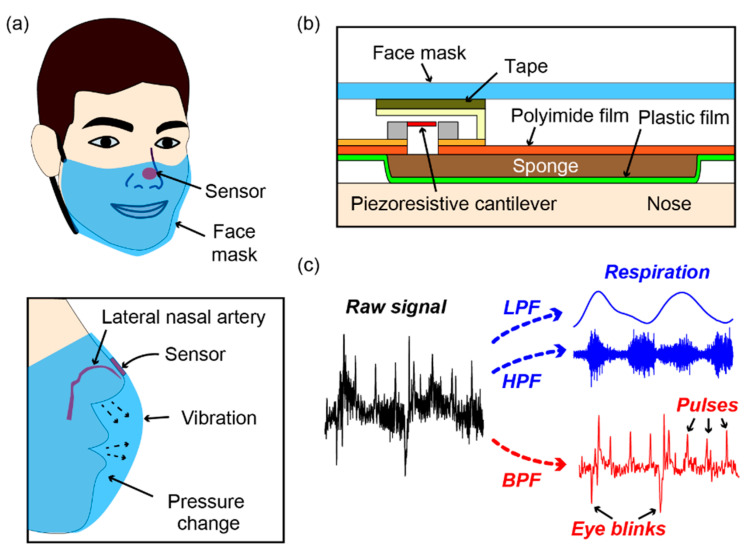
(**a**) Conceptual illustration of the proposed face mask-type sensor. (**b**) Structure of the proposed sensor. (**c**) Sensing principle of the sensor. LPF: low-pass filter, HPF: high-pass filter, BPF: band-pass filter.

To enable simultaneous measurements of pulse waves as well as respiration by both mouth and nose, this paper proposes a face mask-type wearable sensor to monitor these physiological parameters, as shown in Figure 1a. The key element of the sensor is an air bag whose inner pressure change is measured by a highly sensitive microelectromechanical system (MEMS)-based differential pressure sensor, as shown in Figure 1b. The air bag consists of a sponge pad and a cover composed of a plastic thin film and polyimide tape. The MEMS-based differential pressure sensor has a piezoresistive cantilever that deforms when the pressure inside the air bag changes. The cantilever is also deformed by changes in pressure in the surroundings. Therefore, the proposed sensor design enables the measurement of any force, pressure, or vibration acting on the plastic membrane of the air bag as well as to changes in pressure outside the air bag. Figure 1c depicts the sensing principle of the sensor. During measurement, the sensor device is attached to a face mask at the position in contact with the nose of the subject. The respiration of the subject causes pressure changes in the space between the mask and the face of the subject as well as vibrations of the mask. Therefore, the respiration of the subject can be measured by measuring this respiration-induced pressure change and mask vibration. In terms of signal processing, the information about respiration can be extracted from the raw signal of the sensor as the low-frequency component (corresponding to the pressure change) or the high-frequency component (corresponding to the vibration). Moreover, because the sensor is in contact with the nose skin above the lateral nasal artery, it can detect the pulse waves of the subject by detecting the vibrations caused by the arteries. Furthermore, the sensor can detect the eye blinks of the subject by detecting the eye blink-induced relative motion between the facial skin and the mask. We show that this eye blink-induced signal has a pattern that can be distinguished from the pulse wave-induced signal. Therefore, from the sensor signal, it is possible to obtain information related to respiration, pulse waves, and eye blinks by utilizing simple frequency filters and data processing.

This paper reports the design, fabrication, and evaluation of the sensor device and demonstrates the feasibility of simultaneous pulse wave and respiration measurements and eye blink detection using the fabricated sensor.

## 2. Sensor Fabrication

### 2.1. Piezoresistive Cantilever

**Figure 2:**
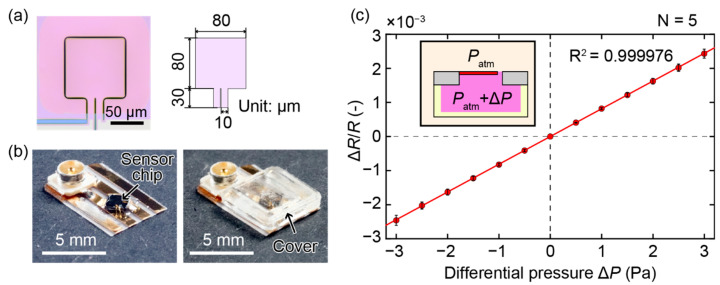
(**a**) Photograph of the fabricated cantilever and design parameters of the cantilever. (**b**) Photographs of the fabricated device with the sensor chip bonded to a printed electrode substrate. (**c**) Relationship between the applied differential pressure and the fractional resistance change of the cantilever.

In this study, we used a 200-nm-thick piezoresistive cantilever [13,14,15,16] as the pressure sensing element. Figure 2a shows a photograph of the fabricated cantilever and its design parameters. The cantilever structure consisted of an 80 μm × 80 μm pad and two 30 μm × 10 μm hinges. The gap surrounding the cantilever structure was 1 μm wide. The sensor chip was attached to a printed electrode board whose thickness was 0.1 mm. The board had a 0.6-mm-diameter through hole that was aligned with the through hole underneath the cantilever of the sensor chip. Then, the electrodes of the cantilever and those of the board were bonded with 30-μm-diameter gold wires. The sensor chip was subsequently covered with a 3D printed cap, which had a hole on the side wall to allow the cantilever to respond to the change in pressure outside the cap. Figure 2b depicts the fabricated device. The dimensions of the 3D printed cap were 5 mm × 5 mm × 0.5 mm and those of the sensor device (not including the part with the connector) were 5 mm × 5 mm × 0.6 mm. The initial resistance of the cantilever was approximately 4.7 kΩ.

To calibrate the cantilever, we applied differential pressure in the range of ±3 Pa to the cantilever and measured the fractional resistance change of the cantilever using a setup described in previous papers [13,14]. The calibration results in Figure 2c clearly show that the fractional resistance change of the cantilever Δ*R*/*R* is proportional to the applied differential pressure Δ*P*. The line in the graph shows the fit of the data, from which we obtained the following relationship between Δ*R*/*R* and Δ*P*: Δ*R*/*R* = *k*Δ*P*, with *k* = (0.81 ± 0.04) × 10^−3^ (Pa^–1^).

### 2.2. Sensor Device

**Figure 3:**
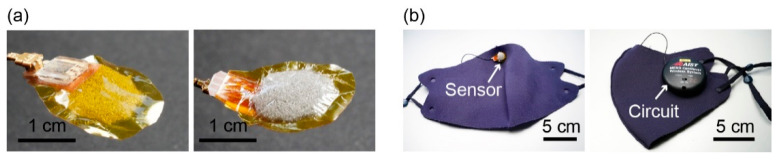
(**a**) Photographs of the fabricated sensor device. (**b**) Photographs showing the sensor device and the measurement circuit attached to a mask.

In a previous paper, we showed that a tube-shaped pressure sensor attached to the nose pad of eyeglasses can detect pulse waves and respiration when a subject wore the eyeglasses [13]. However, we confirmed that when this tube-type sensor is attached to a mask, it cannot detect the pulse waves of the subject wearing the mask. This failure occurs because the pressing force applied to the sensor by the mask is significantly smaller than that when the sensor is pressed against the skin by the nose pad of the eyeglasses. Therefore, to realize the proposed mask-type sensor for simultaneous measurements of pulse waves, respiration, and eye blinks, it was necessary to improve the sensitivity of the sensor so that it could detect the vibrations or motions of the skin caused by these physiological signals when the pressing force applied to the sensor is small. In this study, to improve the sensitivity of the sensor, we proposed using an air bag-shaped structure instead of a silicone tube. In comparison with the tube-shaped structure, the air bag-shaped structure has a larger contact area with the skin, and the thickness of the air chamber shell can easily be reduced to ~10 μm by using thin plastic film, such as food wrap, as the cover. To fabricate the sensor device, a polyester sponge pad (size: Φ15 mm × 2 mm, density: 23 kg/m^3^) was first attached to polyimide tape (thickness: 0.05 mm) with a through-hole (diameter: 2 mm). Then, a 10-μm-thick polyvinylidene chloride film was attached to the tape so that it covered the sponge pad. The sponge pad was used to prevent the air chamber from remaining collapsed due to the sticking of the film to the tape. Finally, the substrate on which the cantilever chip was mounted was attached to the back side of the tape with the through-hole of the cantilever substrate aligned with that of the tape. Figure 3a shows the fabricated sensor device, which was then attached to a face mask as shown in Figure 3b (left). Moreover, we designed and fabricated a circuit to measure the fractional resistance changes of the cantilever and transmitted the data wirelessly to a laptop (Figure 3b (right)). The circuit consisted of a bridge circuit, an amplifier IC (LT6003IS5#TRPBF, Analog Devices Inc., Norwood, MA, USA), an AD converter (LTC2364IMS-16#PBF, Analog Devices Inc., Norwood, MA, USA), a microcontroller (R5F5631MCDFL#V0, Analog Devices Inc., Norwood, MA, USA), and a Bluetooth module (RN42-I/RM, Microchip Technology Japan K.K., Tokyo, Japan), and was powered by a 110 mAh lithium polymer battery (DTP 401525, Shenzhen Data Power Technology Ltd., Dongguan, China). The size of the circuit including the cover was Φ5 cm × 1 cm.

## 3. Measurements and Results

**Figure 4:**
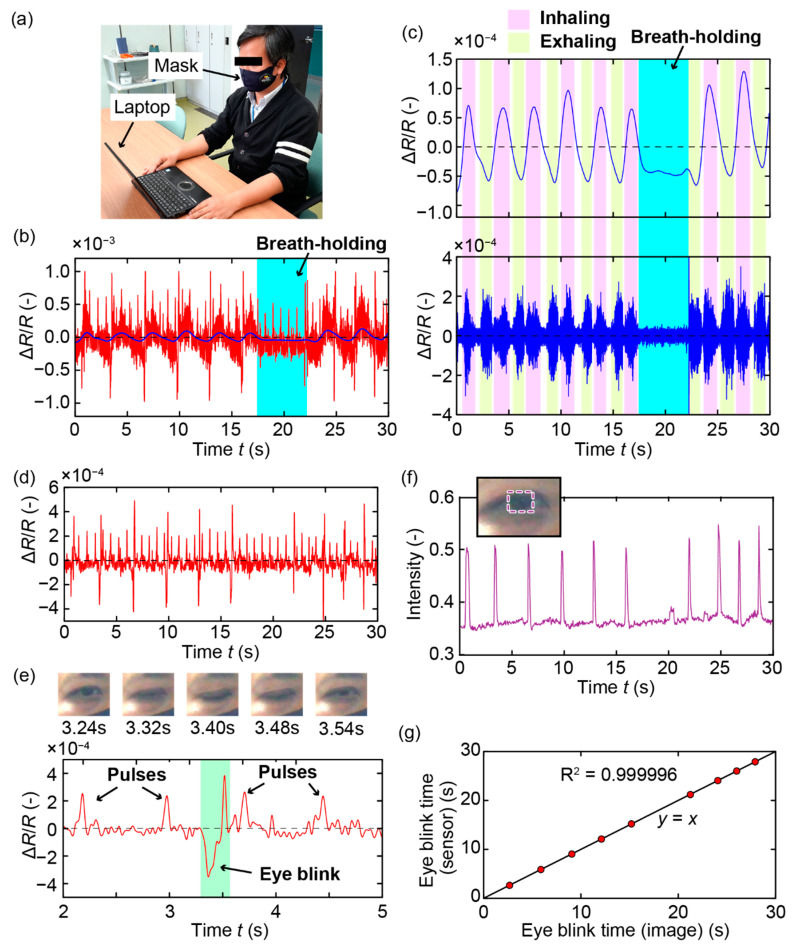
Measurement results in the case of nose breathing. (**a**) Photograph of the measurement setup. (**b**) Sensor raw data (red) and low-pass-filtered data (blue). (**c**) Low- and high-pass-filtered signals showing the respiration of the subject. (**d**) Processed signal showing the pulse and eye blinks of the subject. (**e**) Zoomed-in view of the signal shown in (**d**) and images of the camera showing one eye blinks of the subject. (**f**) Eye blinks of the subject detected from the recorded images. (**g**) Comparison between the timings of the eye blinks obtained from the sensor signal and those obtained from the images.

Using the fabricated sensor device, we demonstrated simultaneous pulse wave and respiration measurements and eye blink detection. Figure 4a shows the measurement setup. A subject (healthy male, 36 years old) was asked to wear the mask and breath normally through his nose or mouth. The circuit attached to the mask measured the resistance change of the cantilever, and the signal was wirelessly transmitted to a laptop PC at a sampling rate of 1000 samples/s. The sensor output was then processed by using Mathematica (version 11.2). Moreover, the eye blinks of the subject were monitored using the camera built into the laptop at a sampling rate of 25 frames/s.

Figure 4b–g presents the results obtained in the case of nose breathing. Figure 4b shows the raw signal of the sensor and the low-pass-filtered (cut-off frequency: 0.3 Hz) signal in red and blue, respectively. During the 30 s of measurements, the subject was asked to hold his breath for approximately 5 s, from *t* = ~17 s to ~22 s, as highlighted in the graph. Firstly, we investigated the ability of the sensor to measure the respiration of the subject. In fact, when the subject inhaled, the pressure in the space between the mask and the face of the subject decreased and the cantilever was pushed outward by the pressure inside the chamber. This bending increased the cantilever resistance, and the tendency of the resistance change is consistent with the calibration results shown in Figure 2b. The lower graph in Figure 4c presents the high-pass-filtered signal of the sensor which corresponded to the vibrations induced by the respiration of the subject. The cut-off frequency was chosen to be 100 Hz because the frequency of the vibration caused by the breath airflow was shown to be in the range of 100–400 Hz [13]. Both the inhalation and exhalation of the subject caused vibrations of the mask that were detected by the sensor. It can be confirmed that these vibrations were induced by the breath as they vanished when the subject held his breath. Furthermore, the inhalation and exhalation of the subject based on the high-pass-filtered signal agree well with the respiration circle obtained from the low-pass-filtered signal. This result indicates that the respiration of the subject could be measured using either the low- or high-pass-filtered signal.

Next, we processed the sensor signal to obtain information about the pulse and eye blinks of the subject. To eliminate the drifts caused by respiration, the raw signal of the sensor (shown in red in Figure 4b) was firstly subtracted by the low-pass signal (shown in blue in Figure 4b). After that, the obtained signal was filtered through a low-pass filter with a cut-off frequency of 10 Hz to eliminate the respiration-induced vibrations. Figure 4d shows the processed signal in which the effect of respiration was significantly reduced. Figure 4e presents the processed sensor signal from *t* = 2 s to 5 s and the images recorded from *t* = 3.24 s to 3.60 s (corresponding to one eye blink of the subject). The graph clearly shows the co-existence of the pulse- and eye-blink-induced signals in the sensor output. Moreover, the pulses caused the cantilever resistance to increase and then return to its original value, whereas the eye blinks caused the resistance to decrease at first, then increase before returning to the original value. Therefore, eye blinks can be detected without the effects of the pulse by detecting the lower peaks of the sensor signal caused by the eye blinks. Then, the pulses of the subject can then be detected after eliminating the signal related to the eye blinks. To confirm the ability of the proposed sensor to detect eye blinks, we compared the results obtained using the sensor signal with those obtained from the images recorded by the camera. The graph in Figure 4f shows the average intensity (mean RGB value) in the image part corresponding to the iris of the subject (the part inside the dashed rectangle in the inset). Upon shutting the eyes, the intensity of this part increased because the upper eyelid appeared brighter than the iris. Therefore, the eye blinks of the subject could be detected from the image series based on the intensity changes, and the times of eye blinks were calculated as the average times at which the intensity was greater than a threshold value (0.4 in this case). From the sensor signal, we calculated the eye blink times by taking the average of the times of the low and high peaks of the signal part caused by the eye blink. Figure 4g compares the eye blink times calculated from the camera images and the sensor signal. Note that the eye blink times were subtracted from the first eye blink times in both the image- and sensor-signal-based data to avoid mis-synchronization between the camera and the sensor signal. The results show that the eye blink times calculated by the sensor are consistent with those calculated from the images.

**Figure 5:**
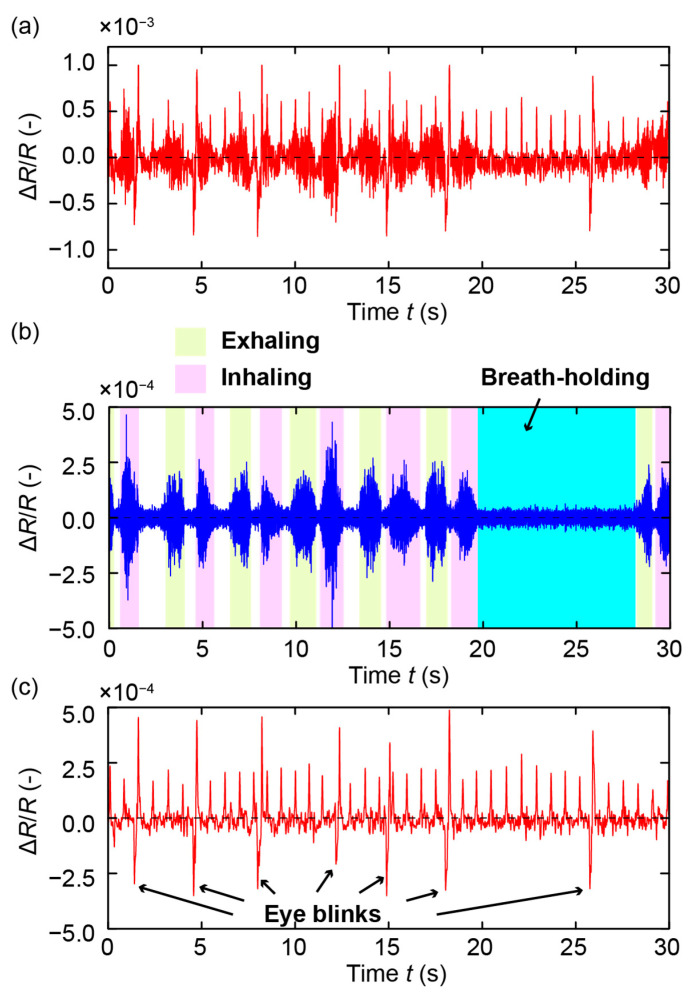
Measurement results in the case of nose breathing. (**a**) Sensor raw signal. (**b**) High-pass-filtered signal corresponding to the respiration of the subject. (**c**) Processed signal showing the pulses and eye blinks of the subject.

We also conducted measurements in the case of mouth breathing. Figure 5a provides the measured raw data. Similar to the nose breathing case, the subject held his breath for about 8 s, from *t* = ~20 s to ~28 s. Figure 5b presents the high-pass-filtered signal (with a cut-off frequency of 100 Hz), where the respiration of the subject can be observed. Similar to the results in the case of nose breathing, vibration appeared in the sensor signal with the inhalation and exhalation of the subject and vanished during breath-holding. Moreover, the pulses and eye blinks of the subject could be measured from the sensor signal in Figure 5c, which is the sensor signal obtained after applying the low-pass filter at a cut-off frequency of 10 Hz. The results demonstrate that the proposed sensor can measure mouth breathing, which could not be measured by the previous eyeglasses sensor.

**Figure 6:**
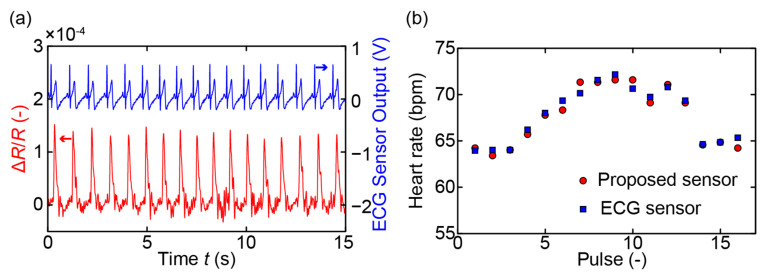
Heart rate measurement. (**a**) The output of the fabricated sensor and the commercial electrocardiogram sensor. (**b**) Heart rates calculated from the outputs of the fabricated sensor and the ECG sensor.

Finally, to confirm the ability of the proposed sensor to measure heart rate accurately, we conducted the simultaneous measurement of the subject’s pulse and electrocardiogram (ECG) using the fabricated sensor and a commercial ECG sensor (AD8232, Analog Devices Inc.), respectively. Figure 6a presents the measured signals of the fabricated sensor and the ECG sensor during the 15 s. Because in this measurement, we focused on the pulses of the subject, the subject was asked to hold his breath and not to blink to eliminate the effects of these parameters on the sensor signal. From the outputs of the proposed sensor and the ECG sensor, we calculated the heart rates of the subject by measuring the intervals of the peaks corresponding to the pulses of the subject. The result shown in Figure 6b indicates that the heart rate calculated from the output of the proposed sensor agreed well with that obtained from the ECG sensor output. In fact, the difference in the heart rate calculated from the output of the proposed sensor and that calculated from the output of the ECG sensor was less than ±1.25 bpm or 1.8%.

## 4. Discussion

In this study, we designed and fabricated a mask-type sensor for simultaneous respiration and pulse wave measurement, and eye blink detection. The sensor consists of a MEMS-based piezoresistive cantilever and an air-bag-shaped air chamber, which has a thin plastic film as the cover. When a force or pressure is applied to the plastic film, the deformation of the film causes the pressure inside the chamber to change, altering the cantilever resistance. We confirmed that by attaching the sensor to a mask at the location in contact with the nose of the subject, the sensor could simultaneously measure the respiration (by either mouth or nose) and pulses of the subject and perform eye blink detection. This simultaneous monitoring was possible because the sensitivity of the sensor was significantly improved by using the air bag-shaped chamber in comparison with the tube-shaped air chamber utilized previously. We showed that respiration could be measured from either the low frequency (below 0.3 Hz) or high frequency (above 100 Hz) component of the sensor signal. Moreover, the pulses and eye blinks of the subject could be observed clearly from the sensor signal after eliminating the frequency components associated with respiration. The pulse- and eye-blink-induced signal patterns are fundamentally different and can be detected separately. The proposed mask-type sensor has a simple structure and signal processing algorithm yet can provide information about multiple physiological parameters. These advantages make the sensor suitable for continuous health monitoring, especially in the current COVID-19 era, in which mask-wearing has become the new normal, as well as in the post-COVID-19 era. Moreover, the proposed sensor is not only useful for measuring heart and respiration rates, which are valuable for detecting and monitoring COVID-19 and other cardiovascular and respiratory diseases, but also for obtaining eye blink information, which is valuable for diagnosing dry eye disease [6], and evaluating drowsiness [5], concentration, and stress.

Although we only reported on the use of the sensor to measure pulse waves, respiration, and eye blinks in this paper, we believe that this sensor can be used to measure other information such as coughing and sneezing, which are also important for diagnosing respiratory diseases such as COVID-19. Moreover, as the sensor can detect the motion of the face skin, it could provide information about emotional facial expressions, which are useful for monitoring mental conditions and in human–computer interactions. In practical applications, the sensor is reusable as it can be easily attached to and detached from the mask.

The commercialization of the sensor should require the development of a packaging method that enables the washing and disinfection of the sensor. Moreover, it should be noted that the measurements in this study were performed on the subject who sat still in a quiet environment. In practical scenarios, it is necessary to reduce the effects of parameters such as the subject’s body movement, voice, ambient sounds, and wind because these parameters also cause the vibration or deformation of the mask resulting in changes to the sensor output. One possible solution is to use two sensors attached to different locations on the mask, for example, one attached inside, and one attached on the outside of the mask, so that both sensors respond to ambient noises but only one sensor responds to the target physiological parameters. Besides, depending on the way the subject wears the mask (for example, tight or loose), the amplitude of the sensor output can vary. Therefore, using masks with adjustable straps will be useful to obtain the optimized wearing. For long-term measurement, the permanent depletion of the sponge can affect the sensitivity of the sensor, which can be solved by reinflating or replacing the airbag.

## References

[1] Koivistoinen T., Lyytikäinen L.-P., Aatola H., Luukkaala T., Juonala M., Viikari J., Lehtimäki T., Raitakari O.T., Kähönen M., Hutri-Kähönen N. (2018). Pulse Wave Velocity Predicts the Progression of Blood Pressure and Development of Hypertension in Young Adults. Hypertension.

[2] Liu X.-N., Gao H.-Q., Li B.-Y., Cheng M., Ma Y.-B., Zhang Z.-M., Gao X.-M., Liu Y.-P., Wang M. (2007). Pulse Wave Velocity as a Marker of Arteriosclerosis and Its Comorbidities in Chinese Patients. Hypertens. Res..

[3] Cruickshank K., Riste L., Anderson S.G., Wright J.S., Dunn G., Gosling R.G. (2002). Aortic Pulse-Wave Velocity and Its Relationship to Mortality in Diabetes and Glucose Intolerance. Circulation.

[4] Chu M., Nguyen T., Pandey V., Zhou Y., Pham H.N., Bar-Yoseph R., Radom-Aizik S., Jain R., Cooper D.M., Khine M. (2019). Respiration rate and volume measurements using wearable strain sensors. NPJ Digit. Med..

[5] Schmidt J., Laarousi R., Stolzmann W., Karrer-Gauß K. (2018). Eye blink detection for different driver states in conditionally automated driving and manual driving using EOG and a driver camera. Behav. Res. Methods.

[6] Divjak M., Bischof H. In Eye Blink Based Fatigue Detection for Prevention of Computer Vision Syndrome. Proceedings of the 11th IAPR Conference on Machine Vision Applications, MVA 2009.

[7] Quer G., Radin J.M., Gadaleta M., Baca-Motes K., Ariniello L., Ramos E., Kheterpal V., Topol E.J., Steinhubl S.R. (2021). Wearable sensor data and self-reported symptoms for COVID-19 detection. Nat. Med..

[8] Jeong H., Rogers J.A., Xu S. (2020). Continuous on-body sensing for the COVID-19 pandemic: Gaps and opportunities. Sci. Adv..

[9] Natarajan A., Su H.-W., Heneghan C. (2020). Assessment of physiological signs associated with COVID-19 measured using wearable devices. NPJ Digit. Med..

[10] Miller D.J., Capodilupo J.V., Lastella M., Sargent C., Roach G.D., Lee V.H., Capodilupo E.R. (2020). Analyzing changes in respiratory rate to predict the risk of COVID-19 infection. PLoS ONE.

[11] Mishra T., Wang M., Metwally A.A., Bogu G.K., Brooks A.W., Bahmani A., Alavi A., Celli A., Higgs E., Dagan-Rosenfeld O. (2020). Pre-symptomatic detection of COVID-19 from smartwatch data. Nat. Biomed. Eng..

[12] Ates H.C., Yetisen A.K., Güder F., Dincer C. (2021). Wearable devices for the detection of COVID-19. Nat. Electron..

[13] Nguyen T.-V., Ichiki M. (2019). MEMS-Based Sensor for Simultaneous Measurement of Pulse Wave and Respiration Rate. Sensors.

[14] Nguyen T.-V., Mizuki Y., Tsukagoshi T., Takahata T., Ichiki M., Shimoyama I. (2020). MEMS-Based Pulse Wave Sensor Utilizing a Piezoresistive Cantilever. Sensors.

[15] Mizuki Y., Nguyen T., Takahata T., Shimoyama I. Highly Sensitive Pulse Wave Sensor with a Piezoresistive Cantilever Inside an Air Chamber. Proceedings of the 2019 IEEE 32nd International Conference on Micro Electro Mechanical Systems (MEMS).

[16] Takahashi H., Dung N.M., Matsumoto K., Shimoyama I. (2012). Differential pressure sensor using a piezoresistive cantilever. J. Micromech. Microeng..

